# Capturing infant and child growth dynamics with P-splines mixed effects models

**DOI:** 10.1038/s41366-026-02112-4

**Published:** 2026-06-12

**Authors:** María Alejandra Hernandez, Zheyuan Li, Tim J. Cole, Yi Ying Ong, Kate Tilling, Ahmed Elhakeem

**Affiliations:** 1 MRC Integrative Epidemiology Unit at the University of Bristol, Bristol, UK; 2 Department of Population Health Sciences, Bristol Medical School, University of Bristol, Bristol, UK; 3 School of Mathematics and Statistics, Henan University, Kaifeng, Henan China; 4 UCL Great Ormond Street Institute of Child Health, London, UK; 5 Yong Loo Lin School of Medicine, National University of Singapore, Singapore, Singapore

**Keywords:** Epidemiology, Risk factors

## Abstract

**Background:**

Investigating early life growth dynamics is important for understanding the developmental origins of obesity. Basis splines (B-splines) provide excellent flexibility for modelling complex growth patterns, but they are prone to overfitting. Penalised B-splines (P-splines) extend B-splines by using a penalty to control their flexibility and avoid overfitting. Despite their advantages, P-splines remain underused in epidemiology, partly due to lack of guidance and accessible software. Our aim was to provide a guide on applying P-spline linear mixed effects models to analyse early life growth trajectories and extract key growth features.

**Methods:**

We outline the theoretical foundation and fitting procedures for P-splines and illustrate their use on repeated height, weight, and body mass index (BMI) measures up to age 10 years from a Southeast Asian birth cohort (*n* = 1014). P-splines linear mixed effects models were fitted by reformulating P-splines as mixed models with sparse matrices for efficient estimation. From the fitted trajectories, we estimated infant peak growth velocity, magnitude and timing of infant peak BMI and childhood rebound BMI, and examined their sex differences, intercorrelations, and associations with prenatal factors.

**Results:**

Infant peak height velocity (means:.4.4 vs. 3.9 cm/month) and peak weight velocity (1121 vs. 890 grams/month) was higher in boys than girls. Infancy peak BMI (17.4 vs. 16.8 kg/m^2^), childhood rebound BMI (15.1 vs. 14.9 kg/m^2^), age at peak BMI (5.8 vs. 6.4 months), and age at rebound BMI (5.4 years) were comparable between sexes. Ages of peak and rebound BMI had a negligible correlation, higher maternal height was associated with higher peak growth velocity, higher maternal early-pregnancy weight was associated with higher and earlier rebound BMI, and higher birth weight was associated with higher and earlier peak BMI.

**Conclusions:**

P-splines simplify knot selection, making them a valuable approach for growth modelling. Software, code and datasets are provided to promote uptake of this method.

## Introduction

Infant and childhood growth trajectories vary between individuals, are primarily shaped by genetic factors and prenatal environments, and can influence subsequent development and disease risk [1]. Modelling repeated measurements over time is key for understanding growth trajectories [2]. Trajectory modelling also enables description of growth features that are vital for understanding development dynamics [3, 4], such as the timing of the childhood body mass index (BMI) rebound which can identify children at increased risk of obesity [5].

Splines are flexible tools for modelling complex growth patterns. They come in various forms, primarily distinguished by their polynomial degree [6, 7]. Conventional regression splines are widely used for longitudinal analyses but can be prone to overfitting because they rely on manual knot selection [6, 8]. P-splines address this by applying a penalty to balance model fit and smoothness [8, 9]. However, despite their advantages and utility for growth chart development [10], use of P-splines for longitudinal growth curve analysis in epidemiology remains limited. This likely stems from perceived complexity and lack of guides and software, highlighting a need for guidance to encourage adoption in epidemiological studies.

This paper addresses this need by providing a guide to using P-spline linear mixed effects models to characterise infant and child growth curves to estimate key growth features. We begin with an overview of the linear mixed effects model. Next, we introduce P-splines as a flexible tool for nonlinear curve fitting, explaining how P-splines extend classic basis splines (B-splines) through penalisation to ensure appropriate smoothing while avoiding excessive flexibility. We then describe the P-spline linear mixed effects model for curve fitting and derivative estimation for growth feature estimation and demonstrate the modelling on data from the Growing Up in Singapore Towards healthy Outcomes (GUSTO) study. Lastly, we discuss practical considerations, alternative modelling strategies, and recommendations for methodological research.

## Nonlinear growth curve modelling with P-splines

### Linear mixed effects (LME) model

The linear mixed effects (LME) model is widely used for analysing repeated measures data. It includes fixed effects to estimate the population-average growth curve and random effects to capture individual-specific deviations from this average and models the repeated outcome as a linear combination of the fixed and random effects [11]. An LME model for a continuous repeated outcome (e.g., weight) as a linear function of a single continuous predictor (e.g., age) that includes random intercepts and random slopes, is written as follows:

1 $${y}_{{ij}}={(\beta }_{0}+{u}_{0i})+{(\beta }_{1}+{u}_{1i})\times {t}_{{ij}}+{\varepsilon }_{{ij}}$$

1.1 $${{(u}_{0i},{u}_{1i})}^{t} \sim N\left(0,{\varOmega }_{u}\right);{\varepsilon }_{{ij}} \sim N\left(0,{\sigma }_{\varepsilon }^{2}\right)$$

where ${y}_{{ij}}$ denotes a single outcome measured in the i-th individual $\left(i=\mathrm{1,2},\ldots ,N\right)$ at age ${t}_{{ij}}\left(j=\mathrm{1,2},\ldots ,{J}_{i}\right)$, with the responses ${y}_{{ij}}$ assumed to be independent between individuals. ${\beta }_{o}$ and ${\beta }_{1}$ are fixed effects that represent the average intercept and slope across all individuals in the population, respectively. ${u}_{{oi}}$ and ${u}_{1i}$ are random effects that quantify how much the intercept and slope for the i-th individual deviate from the population-average intercept and slope, respectively. The random effects are assumed to be normally distributed with mean zero and covariance matrix ${\varOmega }_{u}$. The residual error ${\varepsilon }_{{ij}}$ is assumed to be independently and identically normally distributed with mean zero and variance ${\sigma }_{\varepsilon }^{2}$. Random effects and residual errors are assumed to be mutually independent.

### P-splines LME model

The LME model in Eq. (1) assumes that the outcome changes linearly with age. This model can be extended to capture nonlinear change by replacing the linear age term with nonlinear function of age (e.g., a global quadratic polynomial function for age), with the LME model remaining linear in its coefficients. Among the most flexible tools for modelling nonlinear patterns are splines, which can be integrated into the LME model to replace the linear age term with a curve defined by piecewise polynomials [6, 7].

A widely used and computationally efficient choice is to represent the smooth curve using B-spline basis functions, which provide numerical stability due to their local support and flexible properties [6]. A spline curve expressed in B-spline form is a linear combination of several B-spline basis functions of degree q. Each basis function is a piecewise polynomial defined over q+1 adjacent polynomial segments that are smoothly connected at q specified points called knots. The knots for the full set of basis functions are distributed across the age range, and the combined curve retains continuity of derivatives up to order q−1 at the knots. A common choice is the cubic B-spline which uses degree-3 polynomials (q=3) and gives a smooth cubic spline curve with continuous 1st (velocity) and 2nd (acceleration) derivatives.

The flexibility of a cubic spline is determined by the number (and, to lesser extent, location) of the knots (Supplementary Fig. 1). The number/location of knots must be set manually by the user and can have a significant impact on the B-spline fit. Using too many knots risks overfitting, where the spline becomes excessively ‘wiggly’ by modelling noise alongside the underlying pattern. This reduces generalisability and increases uncertainty [6, 8]. Selecting the optimal knots is a well-known challenge with no single-best solution. A common strategy is comparing models with different knots via information criteria such as Bayesian Information Criterion (BIC). However, this is only valid for Maximum Likelihood (ML) estimation and might not always identify the most appropriate model. An alternative family of functions called penalised splines mitigate these issues and simplify the knot selection process by imposing a roughness penalty on the spline [12].

Penalised B-splines, known as P-splines, introduced by Eilers and Marx [8], are a popular type of penalised splines. They consist of two components: 1) a set of uniformly spaced knots; and 2) a discrete difference penalty of order p, which enforces smoothness by smoothing across adjacent B-spline basis coefficients [8, 9]. This penalty helps prevent overfitting and makes the number of knots less critical. Instead of having to carefully select the number of knots, the user specifies a large approximate basis dimension that will provide sufficient flexibility to capture the underlying trend. The penalty reduces the influence of the final basis size by effectively shrinking the coefficients of the basis functions, allowing the model to find a balance between fitting the data and remaining smooth.

We define the cubic P-spline LME model presented by Djeundje and Currie [13] for the outcome of the i-th subject at the j-th measurement time ${t}_{{ij}}$ as follows:

2 $${y}_{{ij}}=f\left({t}_{{ij}}\right)+{g}_{i}\left({t}_{{ij}}\right)+{\varepsilon }_{{ij}}$$

2.1 $$f({t}_{{ij}})=\mathop{\sum}\limits_{k=1}^{m}{\check{B}}_{k}\left({t}_{{ij}};3\right){\check{\alpha }}_{k}$$

2.2 $${g}_{i}({t}_{{ij}})=\mathop{\sum}\limits_{k=1}^{\widetilde{m}}{\widetilde{B}}_{k}\left({t}_{{ij}};3\right){\widetilde{\alpha }}_{{ki}}$$

where $\left\{{\check{B}}_{k}(;3):1\le k\le m)\right\}$ is a set of m cubic B-spline basis functions representing the population function f (i.e., the fixed effects); $\left\{{\widetilde{B}}_{k}(;3):1\le k\le \widetilde{m})\right\}$ is a set of $\widetilde{m}$ cubic B-spline basis functions representing the individual-specific deviations ${g}_{i}$ (i.e., random effects); and $\check{\alpha }={({\check{\alpha }}_{1},\ldots ,{\check{\alpha }}_{m})}^{\top }$ and ${\widetilde{\alpha }.}_{i}={({\widetilde{\alpha }}_{1i},\ldots ,{\widetilde{\alpha }}_{\widetilde{m}i})}^{\top }$ are two coefficient vectors for the fixed and random B-splines, respectively. To complete the full P-spline specification of the model in Eq. (2), the two p-th order difference penalties for f and ${g}_{i}$ are defined respectively as:

3.1 $${\check{P}}_{\check{\lambda }={\check{\lambda }}_{1}}={\check{\lambda }}_{1}{\check{D}}_{p}^{{\rm{\top }}}{\check{D}}_{p}$$

and

3.2 $${\widetilde{P}}_{\widetilde{\lambda }-\left({\widetilde{\lambda }}_{0},{\widetilde{\lambda }}_{1}\right)}={\widetilde{\lambda }}_{0}I+{\widetilde{\lambda }}_{1}{\widetilde{D}}_{p}^{{{\top }}}{\widetilde{D}}_{p}$$

As we are working with discrete penalties, ${\check{D}}_{p}$ and ${\widetilde{D}}_{p}$ are constructed as *p*-th order difference matrices to discourage large jumps between adjacent basis coefficients. A common choice for enforcing smoothness is the second-order difference (p=2). Allowing different values of p for each smooth term in the model e.g., using p=2 for f and p=1 for ${g}_{i}$, can help reduce collinearity between the population-level and individual-level smoothers [14, 15]. ${\check{\lambda }}_{1}$ and ${\widetilde{\lambda }}_{1}$ are two smoothing parameters that determine the strength of the penalty on the fixed ($\check{\alpha }$) and random (${\widetilde{\alpha }.}_{i}$) basis coefficients, respectively. ${\widetilde{\lambda }}_{0}$ has an additional ridge penalty *I* that addresses identifiability issues [13].

Selecting the optimal value for the smoothing parameter (*λ*) is critical because it controls the weight of the penalty. When *λ* = 0, the fit reverts to the B-spline curve in Eq. (2). Increasing the value of *λ* produces progressively smoother curves by shrinking coefficient differences closer to zero. As *λ* approaches infinity, the fit nears a straight-line estimate. One approach for estimating *λ* is to transform the P-spline into a (linear) mixed effects model [14, 16], where the (unpenalised) bases are expressed as fixed effects components and *λ* as a ratio of variance components. When specified in this way, *λ* can be automatically estimated from the data as part of the model fitting process with methods like restricted maximum likelihood (REML).

The mgcv package in R [12] is a leading tool used for fitting various penalised splines, including penalised splines mixed models. However, mgcv (along with its related function gamm() and the associated gamm4 package) relies on dense model matrices, limiting its ability to efficiently process large datasets (e.g., >1000 individuals). In contrast, the lme4 package [17], widely used for fitting LME models, uses sparse matrices, enabling scalability, but has limited support for incorporating penalised splines. To address this gap, we developed the psme R package [18], which combines the strengths of mgcv and lme4 to efficiently fit the P-spline LME model. Briefly, psme constructs the B-spline basis and penalty matrices in mgcv and reparametrises these matrices into fixed and random effect components, which are then assembled into sparse matrices and fitted as an LME model in lme4.

### Derivative curve estimation

**Fig. 1:**
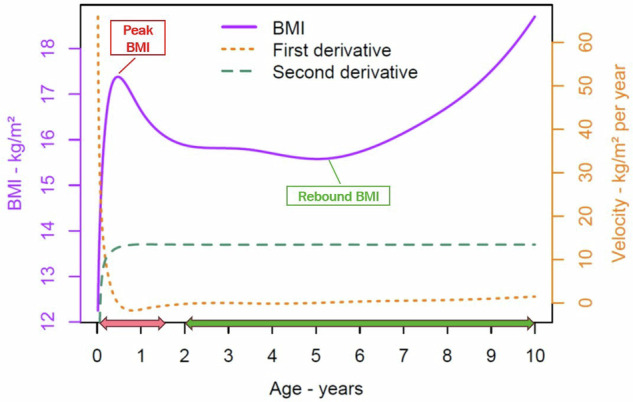
Schematic illustrating infant peak BMI and childhood rebound BMI. Figure shows a typical early life BMI curve and the infant peak BMI and childhood rebound BMI, along with the curve derivatives which will be used to estimate these BMI features from the fitted P-spline LME models. The second derivative is scaled for illustration to allow it to fit in the figure. Infant peak BMI (kg/m^2^) is defined in this study as the highest BMI during the first rise of the BMI curve up to age 18 months. Rebound BMI in kg/m^2^ marks the 2nd rise in BMI after the infant peak and is defined in this study as the lowest BMI after age two years. Peak and rebound BMI can be calculated by identifying the points where the BMI acceleration (second derivative) changes sign. The timings are based on the corresponding ages at peak and rebound BMI, expressed in months and years respectively.

Derivative estimation provides critical insights into growth dynamics by quantifying growth velocity (the first derivative of a growth curve) and acceleration (second derivative) [3, 16]. After fitting a P-spline LME model to repeated anthropometric data, derivatives for the population and individual-specific smoothing functions can be obtained by differentiating the B-spline basis functions and multiplying them by their estimated coefficients. The derivative curves can be used for characterising growth patterns, including peak growth velocity. In addition, derivative turning points (where the 1st or 2nd derivative changes sign) can be used to identify landmarks on the growth curve such as the infant peak BMI and childhood rebound BMI (Fig. 1).

## The Gusto study

### Study design and participants

Pregnant women attending their first trimester antenatal ultrasound scan at Singapore’s two major public maternity units between 2009 and 2010 were invited to participate in GUSTO [19]. Women were eligible if they were aged 18 years or older, Singaporean citizens or permanent residents, with self-reported homogeneous ethnic ancestry (Chinese, Indian, Malay), intended to deliver at either of the recruitment hospitals and reside in Singapore for the next five years. Out of 2034 eligible women invited to participate, 1450 enrolled in the study at 7–11 weeks of gestation. From this group, 1344 women with a singleton, naturally conceived pregnancy were included in the main GUSTO cohort, with 1098 mother-infant pairs remaining in the main GUSTO cohort at the time of delivery.

### Growth measurements

Up to 20 repeated measurements of length/height (cm) and weight (kg) were collected by trained staff during research clinics at approximate ages 3, 6, 9, 12, 15, 18, 24, 36, and 48 months, and 4.5, 5, 5.5, 6, 6.5, 7, 8, 9, and 10 years. Exact age (days) at each measurement was recorded. Crown-to-sole (recumbent) length was measured up to age 24 months (Seca 210 Mobile Measuring Mat) and standing height was measured from age 18 months (Seca 213 Stadiometer). Since both length and height were measured at 18 and 24 months, we prioritised length measurements at these ages, and if length was missing, height was used (*n* = 70 at 18 months; *n* = 74 at 24 months). Weight was measured by calibrated Seca 334 or Seca 803 digital scales. BMI was calculated as weight (kg) divided by height (m²).

Of the initial 1098 infants, 1039 had at least one height and weight measurement between one week and ten years of age (*N* = 15,554 observations). Erroneous values such as duplicates, biologically implausible measurements, carried-forward entries, and extreme outliers were removed using the growthcleanr R package [20], which led to exclusion of 346 observations. Children with only a single measurement were excluded, giving a final growth modelling study sample of 1014 children (48% girls) with at least two repeated measurements of height, weight, and BMI from one week to ten years (*N* = 15,183 observations). Each child contributed a median of 17 (interquartile range: 4) measurements (Supplementary Fig. 2).

### Growth curve modelling

Analyses were performed in R 4.5.0 (R Project for Statistical Computing) and RStudio 2025.09.0 + 387 (Posit Software). The P-spline LME model for age in Eq. (2) was used to construct population-average and individual-specific weight, height, and BMI trajectory curves from age one week to ten years. P-splines were set up as cubic B-splines with evenly spaced knots and bases of size 10 for both the functions *f* and *g*_*i*_. The basis size of 10 is larger than that used in other growth modelling studies [21–23] and should provide sufficient flexibility to capture the nonlinear patterns while remaining computationally efficient. We applied a 2nd order difference penalty on adjacent bases for the fixed effects spline and a 1st order difference penalty on the random spline. Models were fitted separately in boys and girls. A square root transformation was applied to age to better accommodate the rapid growth at younger ages [9, 24]. Models were estimated via REML.

We generated the predicted mean and individual-specific growth curves by evaluating the B-splines on an age grid of 550 points (corresponding to weekly intervals up to ten years) and combining them with the model coefficients. We also generated height and weight velocity curves by calculating the first derivative of the predicted growth curves using the same age grid.

### Estimation of growth curve features

BMI and age at the infant peak and childhood rebound BMI were estimated by identifying trajectory turning points using predicted BMIs up to age 18 months and from age 2 years, respectively (Fig. 1). We first identified the approximate location of turning points by calculating where the smoothed second derivative changes sign: a change from positive to negative indicated a peak and change from negative to positive indicated a trough (rebound). The location of these turning points was subsequently refined by iteratively fitting a quadratic model to an expanding window of neighbouring data points until a well-defined parabolic curve was established. The BMI and age at peak and rebound BMI were calculated from the vertex of this final parabola. This analysis was performed using the getPeakTrough() function from the sitar package [25]. A similar approach was used to estimate the infant peak height and weight velocity from the smoothed first derivatives (up to age 12 months). Lastly, the height and weight velocity curves were used to calculate age-specific infant growth velocity at ages 1, 6, 12, and 24 months.

### Follow-up analysis of estimated growth features

We summarised the distribution of growth features using boxplots and assessed their interrelations with Pearson correlation coefficients. We then used linear regression to examine associations of prenatal maternal and birth factors (maternal age, maternal weight, and maternal height in early pregnancy, and offspring gestational age, birth weight, and birth length) with growth features (peak growth velocity and peak/rebound BMI). Prenatal/birth factors were obtained from maternal reports, clinical measurements, and birth records and these were standardised (mean=0, standard deviation (SD) = 1) prior to analysis. Models were fitted in boys and girls combined with adjustment made for sex.

## Results

**Fig. 2:**
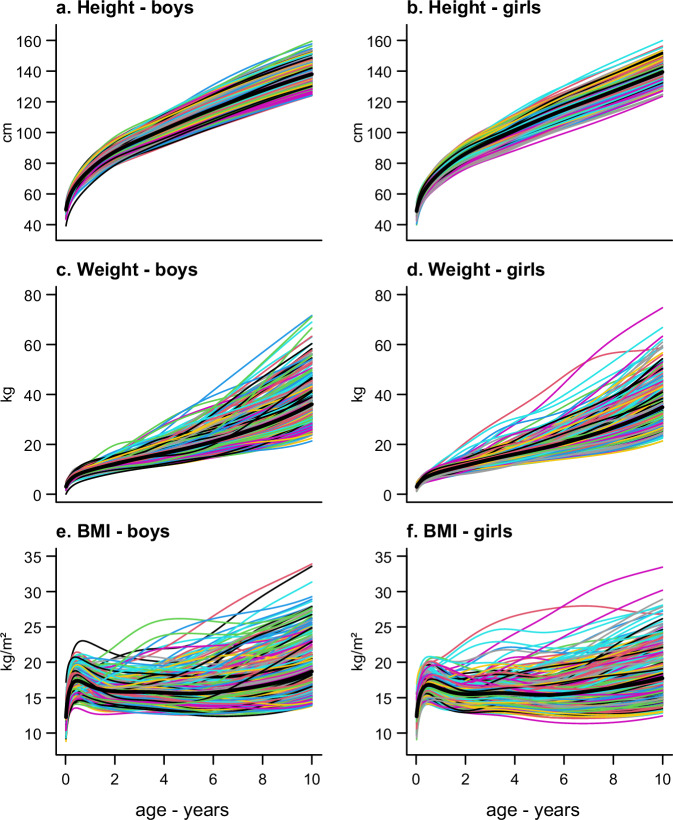
Predicted height, weight, and BMI growth curves in the GUSTO study. Figure shows predicted height (**a**, **b**), weight (**c**, **d**), and BMI (**e**, **f**) growth curves from age one week to ten years from P-spline LME models in GUSTO boys (left panels) and GUSTO girls (right panels). Coloured lines are the individual-specific growth curves; black lines are population-average (mean) growth curves.

Predicted height, weight, and BMI trajectories (Fig. 2) were consistent with the literature [1] and mirrored the observed data for all outcomes with no qualitative difference by the number of repeated measurements (Supplementary Fig. 3). Likewise, the predicted height and weight velocity curves (Supplementary Fig. 4) showed expected rapidly decelerating growth in early life [1].

**Fig. 3:**
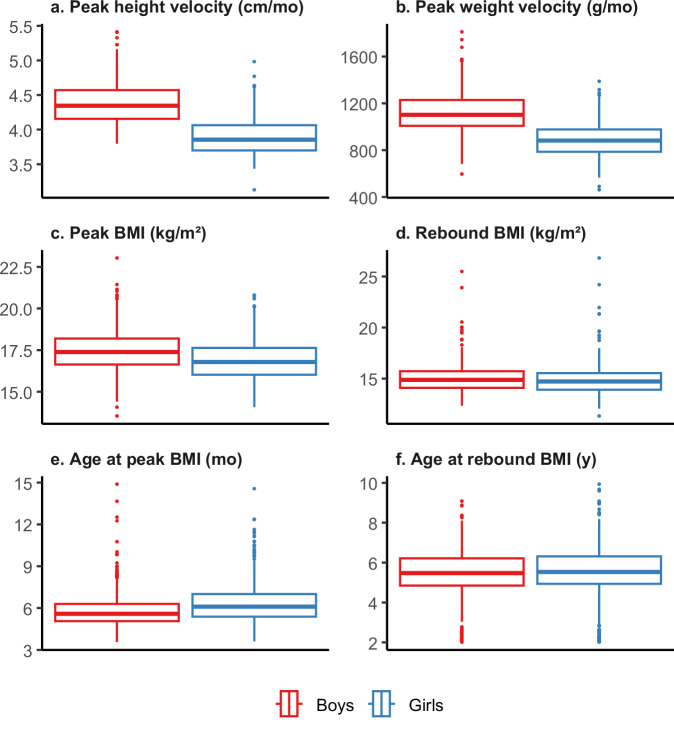
Distribution of estimated infant and child growth features in the GUSTO study. Figure shows box plots summarising the distribution of infant peak height velocity (cm/month) (**a**), infant peak weight velocity (grams/month) (**b**), the magnitude (kg/m^2^) of infant peak BMI (**c**) and childhood rebound BMI (**d**), and the timing (age in months or years) of infant peak BMI (**e**) and childhood rebound BMI (**f**) in boys and girls from the GUSTO cohort. Each box represents the middle 50% of the distribution (i.e., interquartile range). The line inside the box is the median. Whiskers (lines extending from the top and bottom of the box) extend to the minimum and maximum values within 1.5 times the interquartile range. Data points that fall outside the whiskers are plotted as individual points. Means (SD) in boys and girls were 4.4 (0.3) and 3.9 (0.3) [peak height velocity: cm/month], 1121 (181) and 890 (150) [peak weight velocity: grams/month], 17.4 (1.3) and 16.8 (1.2) [peak BMI: kg/m^2^], 15.1 (1.4) and 14.9 (1.5) [rebound BMI: kg/m^2^], 5.8 (1.3) and 6.4 (1.5) [age at peak BMI: months], 5.4 (1.4) and 5.4 (1.6) [age at rebound BMI: years].

Of the 1014 children included in the growth modelling, 109 (10.7%) had no peak or rebound BMI (no peak BMI: *n* = 22, no rebound BMI: *n* = 82, neither: *n* = 5) and 169 (16.7%) had no peak height or weight velocity (no height velocity: *n* = 161, no weight velocity: *n* = 6, neither: *n* = 2). On average, boys (vs. girls) had higher infant peak height velocity (median:.4.3 vs. 3.9 cm/month) and higher infant peak weight velocity (1102 vs. 882 grams/month), but estimates were more similar for infant peak BMI (17.4 vs. 16.8 kg/m^2^), childhood rebound BMI (14.9 vs. 14.7 kg/m^2^), age at peak BMI (5.6 vs. 6.1 months), and age at rebound BMI (5.5 years). (Fig. 3). These estimates were largely consistent with the literature [5, 26], including with estimates obtained using different methods in GUSTO [21, 27]. There was also considerable variability in growth features between children (Fig. 3). For example, the SD around the mean peak height velocity in boys (mean: 4.4 cm/month) was 0.3 cm/month, and the SD of mean age at rebound BMI in girls (mean: 5.4 years) was 1.6 years (see caption in Fig. 3 for all mean and SD values).

**Fig. 4:**
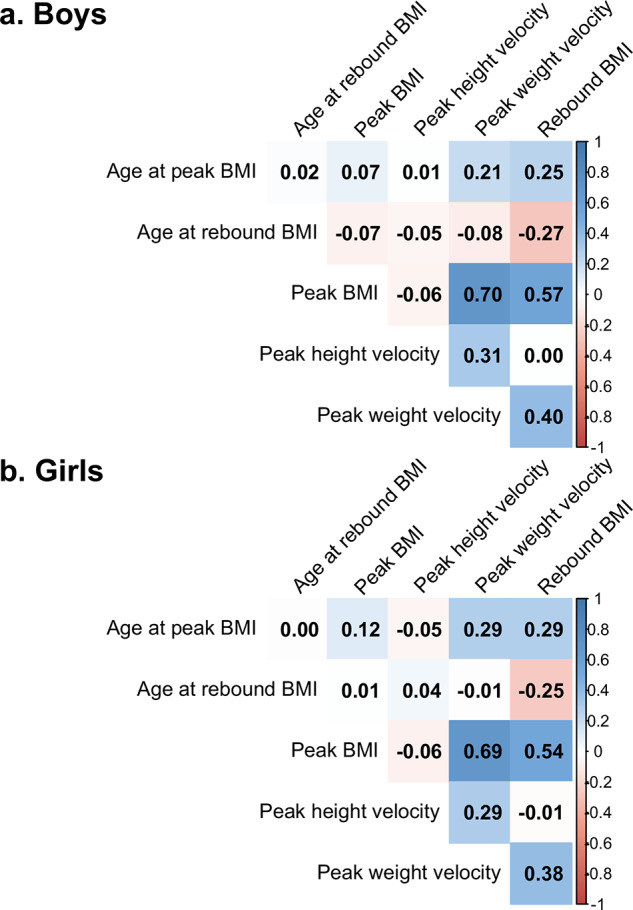
Growth feature correlations in the GUSTO study. Figure shows correlation matrices for infant peak height/weight velocity and magnitude and timing of the infancy peak BMI and childhood rebound BMI, separately in boys (**a**) and girls (**b**) from the GUSTO study. The numbers represent Pearson correlation coefficients for pairs of growth features.

**Fig. 5:**
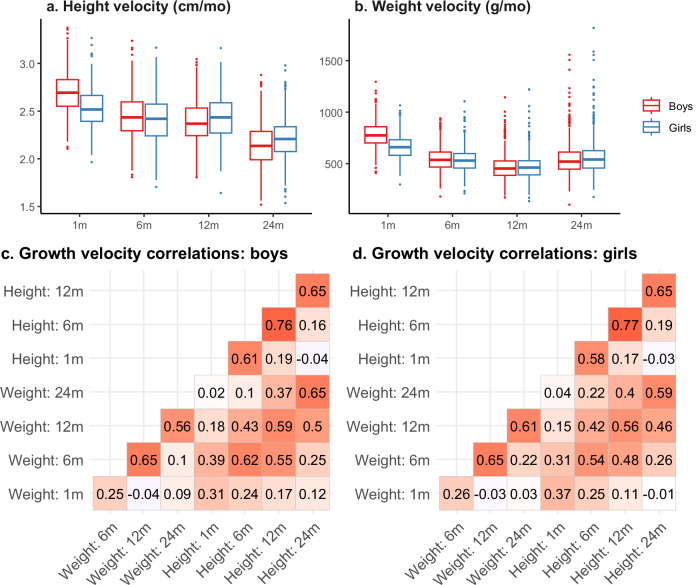
Distribution and correlations of infant height and weight growth velocity at age 1, 6, 12, and 24 months in the GUSTO study. Figure shows box plots summarising the distribution of infant height velocity (cm/month) (**a**) and weight velocity (g/month) (**b**) at ages 1, 6, 12, and 24 months, and their correlations in boys (**c**) and girls (**d**) in GUSTO. Each box (in (**a**, **b**)) represents the middle 50% of the distribution (i.e., interquartile range). The line inside the box is the median. Whiskers (lines extending from the top and bottom of the box) extend to the minimum and maximum values within 1.5 times the interquartile range. Data points that fall outside the whiskers are plotted as individualpoints. The numbers in (**c**, **d**) represent Pearson correlation coefficients.

Pairwise correlations between peak height/weight growth velocity and peak/rebound BMI features were virtually identical in boys and girls (Fig. 4). These included a negligible association between the ages of peak and rebound BMI (*r* ≤ 0.02), a low negative association between the BMI and age at rebound BMI (*r* ≤ −0.27), low positive association between peak height and weight velocity (*r* ≤ 0.31), and moderate positive associations between peak and rebound BMI (*r* ≤ 0.57), and between infant peak weight velocity and peak BMI (*r* ≤ 0.70). Boxplots and pairwise correlations for age-specific growth velocity (at age 1, 6, 12, and 24 months) showed that growth velocity for both height and weight was highest at age 1 month and that inter-age correlations diminish as the time interval increases, until reaching almost no association between age 1-month and 24-month growth velocity (Fig. 5).

**Fig. 6:**
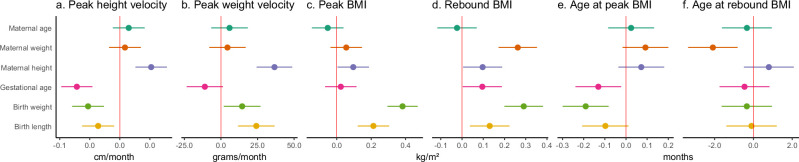
Association of maternal prenatal and birth factors with infant and child growth features in the GUSTO study. Figure shows mean differences (points) and 95% confidence intervals (horizontal bars) in infant peak height velocity (**a**) and peak weight velocity (**b**), the magnitude of the infant peak BMI (**c**) and childhood rebound BMI (**d**), and the ages at peak BMI (**e**) and rebound BMI (**f**) per a 1-unit standard deviation unit higher maternal age, maternal weight, maternal height, and offspring gestational age, birth weight, and birth length in the GUSTO study. Models included boys and girls and were adjusted for sex. Mean differences in age at rebound BMI are shown in months to aid presentation. The analysis sample comprised 823 children with complete data on all variables.

Associations of maternal prenatal and birth factors with the estimated infant and child growth curve features (peak growth velocity and peak/rebound BMI) are presented in Fig. 6. Among maternal factors, higher maternal height was associated with a higher peak height and weight velocity, and higher peak and rebound BMI. Higher maternal weight was associated with higher and earlier rebound BMI, whereas maternal age was not associated with any outcomes. For example, mean difference in childhood rebound BMI per SD unit higher maternal weight was 0.26 kg/m^2^ (95% CI: 0.17–0.35). Among birth factors, higher gestational age was associated with lower infant peak height velocity, a younger age at peak BMI, and a higher rebound BMI. Higher birth weight and birth length were associated with lower peak height velocity, higher peak weight velocity, and higher peak and rebound BMI. Higher birth weight also associated with younger age at peak BMI. For example, the mean difference in peak height velocity per SD unit higher gestational age was −0.69 cm/month (−0.93 to −0.44).

## Discussion

### Summary

We have introduced the P-spline LME model and demonstrated its application to early life growth trajectories in a South Asian birth cohort. P-splines address the challenge of manual knot selection in conventional regression splines by combining B-splines (with evenly spaced knots) with a penalty term that mitigates overfitting. Our fitted trajectories closely matched the observed data and existing literature, supporting validity of the P-spline LME approach. We also showed how curve derivatives can be estimated, highlighting the model’s utility in deriving important growth metrics that can provide novel insights into developmental processes. For example, the low-moderate correlations between peak growth velocity and BMI features suggest that they may each be individually useful for identifying genetic and environmetnal mechanisms of obesity development. Likewise, the negligible correlations between growth velocity at ages 1 and 24 months suggests that growth during the first month may be more strongly determined by intrauterine environments, and that genetics become more influential later in infancy to guide children towards their predetermined growth trajectory, which supports the concept of homeorhesis (or canalisation) [1].

### Practical considerations

Our psme package reformulates P-splines as mixed models with sparse fixed and random effects to enable efficient estimation. It took only 34 seconds to fit the six P-spline LME models, highlighting the psme package’s efficiency and scalability for larger datasets. The mixed model representation also supported REML (or ML) estimation of the smoothing parameters. We chose REML instead of ML estimation because ML estimates of variance components tend to be biased downwards, which can lead to oversmoothing [28]. REML is also preferred over generalised cross-validation because it tends to produce smoother fits [29].

We used 1st order differences penalty for the random spline so that it is not collinear with the population spline (2nd order). The choice of penalty order can impact the flexibility and shape of the fitted curve and so it is worth exploring different options [9]. The penalty in psme follows Djeundje and Currie [13] but alternatives have been proposed [29]. One such method adds a second difference penalty to both the population and individual-specific curves to improve derivative estimates [30]. Applying this double penalty to GUSTO, and optimising smoothing parameters using the Separation of Overlapping Precision Matrices method [16, 31], showed that the growth features derived from the double penalty approach were strongly correlated with those from our psme penalty method (Supplementary Fig. 5).

Following the classic P-spline approach of Eilers and Marx [8], we selected a B-spline basis rather than alternatives such as truncated power functions [32], which are considered inferior due to poor numerical conditioning and an inflexible penalty structure [33]. Using 10 bases provided good flexibility and computational efficiency and should be sufficient for most applications in longitudinal trajectories in epidemiological studies. It is generally impossible, at least theoretically, to have too many B-splines because the penalty rather than the number of knots controls the smoothness. However, increasing basis sizes comes with computational cost. Further, we used the same basis size for both population and random splines but studies with fewer repeated measurements may need to use a smaller basis in the random spline.

Transforming the age covariate can improve model fit by linearising relationships [9, 24], and in our example, a square root transformation of age produced more suitable curves than either the original age scale or a log transformation. Transforming the repeated outcome can help address skewness and heteroscedasticity to ensure residuals better meet model assumptions, but such transformations alter the scale and interpretation of the estimated parameters. Lastly, it is worth noting that 10.7% of the included GUSTO participants had no identifiable peak or rebound in BMI, which may reduce the precision of estimates in subsequent analyses and their exclusion can distort associations with later outcomes [34].

### Alternative modelling approaches

Numerous alternative methods exist for modelling growth trajectories. Functional principal component analysis, for example, estimates the mean trajectory using a functional basis and captures individual-specific deviations through principal components [35], often smoothing mean and covariance functions with P-splines [36] or local polynomials [37]. Another widely used method, SITAR (Super Imposition by Translation And Rotation), models a mean curve with a natural spline and summarises individual-specific curves using up to 4 random effects [38–40]. LME models with fractional polynomials can also be used [41, 42] as can global polynomials [43] though the latter lack the local support found in splines. Linear spline LME models are also commonly used, and while they do not produce smooth curves, they provide interpretable slope estimates for specified age windows [7, 44]. At the group level, clustering methods like latent trajectory models can identify population subgroups with different trajectories [7, 45]. More recently, approaches that integrate machine and deep learning within mixed effects and functional data frameworks are emerging as promising for prediction tasks [46–48]. Finally, P-splines can also be implemented within a Bayesian framework [49].

### Recommendations for methodological research

Research is needed to systematically evaluate P-spline LME models against other approaches to growth curve analysis and in cohorts with differing numbers of repeated measures, sample sizes, age ranges, and patterns of missing data. Comparative studies are also needed to assess the impact of different P-spline model specifications, including alternative age and outcome transformations and penalty structures. Studies that demonstrate the utility of P-splines for modelling pooled individual-level data from multiple cohorts, and for modelling outcomes beyond physical growth such as mental health score trajectories, are also warranted.

## Conclusions

P-spline LME models are a valuable method for characterising nonlinear growth curves in longitudinal population studies. A new R package (psme), R scripts, and synthetic datasets are provided to support uptake of this method. We hope this resource enables epidemiologists to apply the method in their own longitudinal studies.

## Supplementary information

Supplementary material

## Data Availability

The data that were used in this study are described in https://gustodatavault.sg. Interested researchers can request access to the GUSTO data by submitting a research proposal for assessment by the GUSTO Executive Committee, who will then and release the data. All R code used in this paper and a synthetic copy of the GUSTO dataset can be found on GitHub at https://github.com/LongitudinalModelling/psplines. The synthetic GUSTO datasets are provided to allow interested readers to practice the methods and were generated from P-spline LME model predictions via the synthpop package in R.
