# Supplementary material for: Capturing infant and child growth dynamics with P-splines mixed effects models
Source: Int J Obes (Lond). 2026 Jun 12;50(8):1809–17. doi: 10.1038/s41366-026-02112-4 (PMC13461364; doi:10.1038/s41366-026-02112-4)
Supplement: Supplementary file 1 [file 41366_2026_2112_MOESM1_ESM.docx]

**Supplementary Figure 1.** Cubic B-spline construction and the impact of basis size on spline flexibility.

Figure shows two cubic B-splines with evenly spaced knots fitted to the same simulated noisy data (grey points). The left panel (a) uses 7 basis functions (3 internal knots) and the right panel (b) uses 21 basis functions (17 internal knots). The fitted splines are shown in black. Coloured curves represent the individual cubic B-spline basis functions, each scaled by its fitted coefficient from the linear model, which together form the spline. The small black ticks across the horizontal axis indicate the locations of the knots, which are the points where the spline's polynomial segments are smoothly joined. Red points on the spline mark its value at the x-coordinate where each corresponding basis function reaches its peak, marking the region of strongest influence of that basis function on the spline. Comparing panels illustrates that increasing basis size provides greater local control and pattern capture but also increases the risk of overfitting by capturing random noise instead of underlying trends.

**Supplementary Figure 2.** Observed height, weight, and BMI measurements in the GUSTO study.

Figure shows the 15,183 longitudinal observations of height, weight, and BMI from 1,014 GUSTO boys and girls that were used for P-spline LME analysis. The data span from age one week to 10 years and each individual had a minimum of two repeated measurements.

**Supplementary Figure 3.** Predicted versus observed weight, height, and BMI for a randomly selected boy and girl with respectively 5, 10, and 15 repeated growth measurements.

Figure shows predicted growth curves (from P-spline LME models) versus the observed values (circles) for three boys and three girls that were randomly selected according to the number of repeated measurements (5, 10, or 15).

**Supplementary Figure 4.** Predicted weight and height velocity curves.

Figure shows the individual-specific height and weight growth velocity curves from 1 week to 10 years in GUSTO boys and girls (i.e., the first derivative of the predicted growth curve).

**Supplementary Figure 5.** Comparison of growth features estimated from our P-spline models (psme) with estimates from P-spline models using the double penalty specification.

Figure shows scatterplots and Pearson correlation coefficients (r) between growth features estimated from P-spline LME models that use the penalty method in the psme package in R (i.e., the models used in this paper) and P-spline LME models using the double penalty method described in Hernandez et al 2023. The red lines are lines of equality. Data are shown for GUSTO boys.
